# Detection of Inflammatory and Homeostasis Biomarkers after Selective Removal of Carious Dentin—An In Vivo Feasibility Study

**DOI:** 10.3390/jcm10051003

**Published:** 2021-03-02

**Authors:** Jana Schmidt, Clemens Hübler, Sandra Krohn, Gerhard Schmalz, Hartmut Schneider, Thomas Berg, Rainer Haak, Dirk Ziebolz

**Affiliations:** 1 Department of Cariology, Endodontology and Periodontology, University of Leipzig, Liebigstr. 12, 04103 Leipzig, Germany; Clemens.Huebler@medizin.uni-leipzig.de (C.H.); gerhard.schmalz@medizin.uni-leipzig.de (G.S.); hartmut.schneider@medizin.uni-leipzig.de (H.S.); rainer.haak@medizin.uni-leipzig.de (R.H.); dirk.ziebolz@medizin.uni-leipzig.de (D.Z.); 2 Division of Hepatology, Department of Medicine II, University Hospital Leipzig, Liebigstr. 20, 04103 Leipzig, Germany; sandra.krohn@medizin.uni-leipzig.de (S.K.); thomas.berg@medizin.uni-leipzig.de (T.B.)

**Keywords:** carious dentin, selective carious tissue removal, inflammatory cytokines, matrix metalloproteinases, diagnostics of pulp inflammation

## Abstract

Deep carious dentin lesions induce an immune reaction within the pulp-dentin complex, leading to the release of cytokines, which might be suitable biomarkers in pulp diagnostics. This in vivo feasibility study determines the concentration of different cytokines after selective removal of carious infected dentin (SCR). In our methodology, paired samples are obtained from 21 patients—each of them with two deep carious lesions at posterior teeth without clinical symptoms. After SCR, lesions are randomly assigned to treatment strategy: Group 1 (11 patients): Carious dentin is covered either with Biodentine^TM^ (*n* = 11) or gutta-percha (*n* = 11) before using the adhesive Optibond^TM^ FL. Group 2 (10 patients): The adhesives Clearfil^TM^ SE Protect Bond (*n* = 10) or Clearfil^TM^ SE Bond 2 (*n* = 10) are directly applied. Prepared cavities are rinsed with phosphate buffered saline containing 0.05% Tween 20 (10X) for five minutes immediately after SCR (visit 1) and eight weeks later (visit 2). Rinsing liquid is regained. Concentrations of IL-1β, IL-6, IL-10, C-reactive protein (CRP), TNF-α, IFN-γ, TIMP-1, -2, and MMP-7, -8, -9 are assessed by customized multiplex assays, evaluated with fluorescence analyzer. Non-parametric statistical analysis (Wilcoxon, Mann–Whitney U Test, *p* < 0.05) is performed (SPSS 25). Our results show that concentrations of CRP, IL-1β, IL-6, TIMP-1, -2, and MMPs were detectable. Median concentrations of CRP, IL-1β und IL-6 were significantly higher in visit 1 (304.9, 107.4, 3.8 pg/mL), compared to visit 2 (67.8, 2.3, 0.0 pg/mL; p_i_ < 0.001). The study revealed that the non-invasive determination of cytokines from prepared dental cavities is possible.

## 1. Introduction

Dental caries is a multifactorial disease that is associated with an imbalance of the oral microflora and local environmental factors promoting pathogenic acid-producing bacteria resulting in demineralization processes at an early stage and degradation of organic matrices at an advanced stage of disease [1,2]. When the carious lesion reaches the dentin, bacteria, as well as their metabolic products, interact with pulpal cells via dentin tubules, and the pulp-dentin complex reacts with immunological defense mechanisms, releasing pro-inflammatory, as well as anti-inflammatory cytokines [3,4]. When dentin carious lesions progress into the inner dentin zone, the risk of pulp injuries by therapeutic intervention is growing [5,6]. As described above, several defenses and regeneration mechanisms exist mediated by odontoblasts, which release antimicrobial peptides and cytokines, thus initiating the migration of immunocompetent cells. Furthermore, an increase of cytokines was found in pulp cells (e.g., transforming growth factor β (TGF-β), interleukin-1β (IL-1β), interleukin-8 (IL-8), interleukin-6 (IL-6), interleukin 10 (IL-10), interferon-γ (IFN-γ), and tumor necrotic factor-α (TNF-α) [3,7,8]. A current systematic review summarized different studies considering several of these cytokines as markers in the detection of pulp inflammation, comparing healthy pulps with irreversibly inflamed pulps [9]. Most studies focused on pulp tissue and blood samples [9]. Another review pointed out that the current data availability is poor and more studies are needed [4]. The inflammatory cytokines and chemokines may also be detectable in dentin carious lesions close to the pulp and not only in sample material gained from the pulp [10].

Concerning the self-healing and reparative potential of the pulp in the case of given pulpal immunocompetence, it seems reasonable not to expose the pulp during carious tissue removal, but to leave caries infected dentin in deep dentin lesions without clinical symptoms of irreversible pulpitis or pulp necrosis. This therapeutic approach called selective caries therapy (SCT) has been shown to be advantageous compared to non-selective carious tissue removal in deep dentin caries lesions [5,11,12].

SCT means leaving different zones of dentin. That might have an impact not only on the adhesive sealing, but also on the penetration of the adhesive into the pulp. Adhesive restoration means principally applying cytotoxic chemicals that might diffuse through dentin tubules, causing immunological reactions and inflammatory responses [13,14,15,16]. In demineralized zones with a higher width of dentin tubules, a greater penetration depth of the adhesive can be assumed, whereas the deposition of whitlockite and other minerals as intratubular crystals [17,18] may reduce the diffusion of adhesive. Furthermore, pulpal hydrostatic pressure limits adhesive components’ access in the direction of the pulp [19].

Materials have been developed and studied (e.g., cements based on calcium hydroxide or calcium silicate), which have a high pH value and aim at preserving pulp vitality, mainly by promoting reactionary dentine formation [20,21,22]. Thus, they may induce a shift of the inflammatory response within the pulp towards healing. Biodentine^TM^ (Septodont, Saint-Maur-des-Fossés Cedex, France, BD) is such a calcium silicate cement with favorable properties (e.g., antibacterial activity [23], biocompatibility [24,25]). The technique of immediate adhesive restoration after placement and setting of BD (12 min [26]), as applied in group 1 of the current study, is supported in recent literature [27,28]. Considering the direct application of dental adhesives on residual carious dentin after SCT in deep cavities, conflicting results with regard to the influence on pulpal cells are existing [29,30,31]. Several studies showed that adhesives are biocompatible and do not induce clinically relevant irritation of pulpal cells in deep dentin lesions with intact dentin barrier to the pulp [13,32,33,34]. Furthermore, for several dental adhesives, e.g., Clearfil^TM^ Protect Bond (Kuraray Noritake Dental Corporation, Okayama, Japan; PB), but also Clearfil^TM^ SE Bond (Kuraray Noritake Dental Corporation, Okayama, Japan; SE), antibacterial efficiency has been shown [35,36]. Thus, the additional therapeutic benefit of applying pulp protective materials in this clinical situation is worth being further elucidated.

Besides the cellular impact and consideration of pulpal inflammation, leaving carious altered dentin might also affect the adhesive bonding of composites. Cytokines, such as matrix metalloproteinases (MMP), interleukins (IL), and tissue inhibitor of metalloproteinases (TIMP), have been investigated with regard to the strength and durability of adhesively luted (composite) restorations [37].

The present feasibility study aimed to investigate the concentration of different pro-inflammatory cytokines, as well as tissue homeostasis proteins in the cavity immediately after SCT and eight weeks after the restoration of the cavities with different materials.

As the main question of the present study, it was hypothesized that the concentration of the analytes can be determined in rinsing solutions from cavities of deep dentin carious lesions. Subsequently, two auxiliary questions were addressed: Firstly, pro-inflammatory cytokines’ concentration was supposed to be higher immediately after SCT than eight weeks later. Secondly, it was hypothesized that covering the remaining carious dentin with a tricalcium silicate cement leads to lower concentrations of pro-inflammatory cytokines during re-entry after eight weeks compared to the direct application of self-etching adhesive.

## 2. Materials and Methods

### 2.1. Origin of Samples: Study Design, Patient Recruitment, and Randomization

The sample material for analyses within this feasibility study was obtained during the course of two recently published in-vivo studies [23,35]. Both of them were two-arm blinded interventional clinical studies approved by the ethics committee of the local Medical Faculty (ethics committee vote number 010-15-26012015, 368-15-05102015) and registered in the German Clinical Trials Register. Overall, twenty-one patients were included in the present investigation according to the criteria given in the consort diagram (Supplementary Figure S1). Each study participant presented two posterior teeth (twenty-eight molars, fourteen premolars in total) with deep primary carious lesions (radiographic extent ≥ R3b). Study participants gave written informed consent after being informed verbally and in writing about the aim of the respective study and its course. The first study (group 1) started in March 2016 and ended in May 2017. Group 2 was recruited within the second study between January 2017 and April 2018.

**Figure 1:**
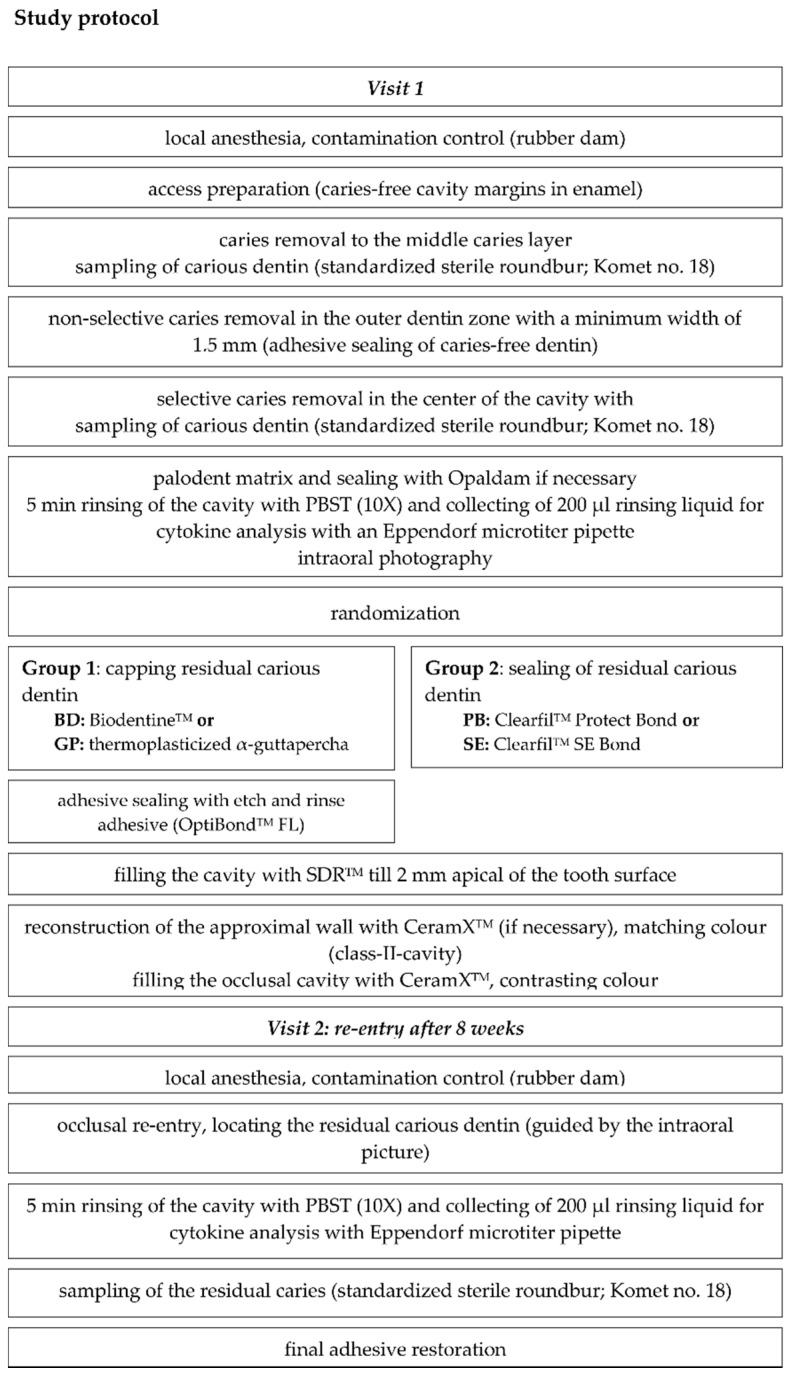
Flowchart of study performance. Please find detailed information considering the used materials in Table S1. PBST: phosphate buffered saline containing 0.05% Tween 20.

Detailed descriptions of study performance and dental therapy, as well as a restorative treatment, are given in the respective publications [23,35]. The flowchart of both studies is summarized in Figure 1.

In both studies, the clinical performance was randomized by drawing lots. For each patient, two lots (singed with PB: Clearfil^TM^ Protect Bond and SE: Clearfil^TM^ SE Bond 2 or BD: Biodentine^TM^ and GP: Guttapercha as control, respectively) were assigned to the study teeth by the study assistant. There was no randomization according to a criterion.

### 2.2. Sample Processing and Cytokine Analysis

Bead-based immunoassays were used for cytokine analysis in samples according to the manufacturer’s recommendations. IL-1β, IL-6, IL-10, C-reactive protein (CRP), TNF-α, IFN-γ, TIMP-2, and TIMP-1 were quantified with a customized LegendPlex Panel (BioLegend GmbH, Koblenz, Germany). For MMP-7, -8, and -9, a Human Custom 3-Plex assay (Aimplex Biosciences, Inc., Pomona, CA, USA) was used. Both of them are classical ELISA immunoassays using capture and detection antibodies.

The assays were performed according to the manufacturer’s recommendations. A detailed description of laboratory processes is given in Table S2. 50% of samples (*n* = 37) had to be diluted with phosphate buffered saline containing 0.05% Tween 20 (10X; abbreviated PBST) for reaching the required volume. Dilution factors were considered when calculating the concentrations where necessary.

Fluorescence intensity on the beads was quantified using a BD LSR II flow cytometer (BD Biosciences, San Jose, CA, USA). Data of flow cytometry were analyzed with the program BD FACS Diva 8.1 (BD Biosciences). For MMP-7, -8, and -9, 300 events were considered per bead, whereas 1500 events were measured for the other beads. The detection limits were as follows: IL-1β—0.82 pg/mL, IL-6—1.76 pg/mL, IL-10—1.15 pg/mL, CRP—10.28 pg/mL, TNF-α—1.48 pg/mL, IFN-γ—1.99 pg/mL, TIMP-1—127.13 pg/mL, TIMP-2—20.78 pg/mL. Results with maximal fluorescence intensity lower than Standard 1 (0.00 pg/mL) in the Aimplex assay are reported as “not detectable”.

### 2.3. Statistical Analysis

For testing the normal distribution of data at the different intervention times, the Shapiro-Wilk test was applied with a significance level of α = 0.2. Only TIMP-1 concentrations showed normal distribution except for visit 1 in group 1 (BD and GP). Non-parametric testing by the Wilcoxon test and Mann–Whitney U test was performed. Testing was carried out exploratively, due to the character of the present investigation as a feasibility study. α = 0.05 was considered to be statistically significant. Due to the exploratory nature of this study, the raw *p*-values were indicated, a correction due to multiple testing was not carried out. Changes in analyte concentrations from visit 1 to visit 2 were investigated irrespective of the intervention group by pooling “all groups”, resulting in a higher number of samples. Furthermore, in a second step, the changes within the single groups were considered.

## 3. Results

### 3.1. Demographic and Clinical Characteristics of Study Participants and Teeth

Twenty-one clinically symptom-free patients, and subsequently, 42 teeth (twenty-eight molars, fourteen premolars) were included in both studies, as recently published [23,35]. The participants’ demographic and clinical characteristics, as well as the teeth’s properties, are given in Table 1. Forty cavities were class II (95%), and two were class I (5%) in total. One study participant (age of 39 years) of study group 2 (SE) reported postoperative symptoms of an irreversible pulpitis one week after visit I at the SE tooth. No other reasons for drop-outs occurred during both of the studies.

Sample collection was not possible in five cavities at visit 1 because the cavities could not be sealed properly with matrix system and Opaldam (Ultradent Products, South Jordan, UT, USA), leading to loss of rinsing fluid. At visit 2 two samples are missing in group 2, due to drop-out and the above-mentioned sampling problems, respectively. Due to laboratory processes, analysis of MMP-7, -8, and -9 was not performed in two patients of group 1 at visit 2 (Figures S1 and S2).

### 3.2. Pro-Inflammatory Cytokines

IL-1β, IL-6, CRP, TIMP-1, -2, as well as MMP-7, -8, and -9, were found in concentrations above the detection threshold of the respective assay and could be further analyzed. IL-10 and TNF-α concentrations were below the detection limit of this custom assay in all samples. Considering INF-γ in the GP/BD group, 10 out of 22 samples at visit 1 and one out of 23 samples at visit 2, respectively, showed detectable concentrations. In the PB/SE groups, one out of 15 at visit 1 and two out of 17 samples at visit 2 were above the detection threshold of 1.99 pg/mL.

**Figure 2:**
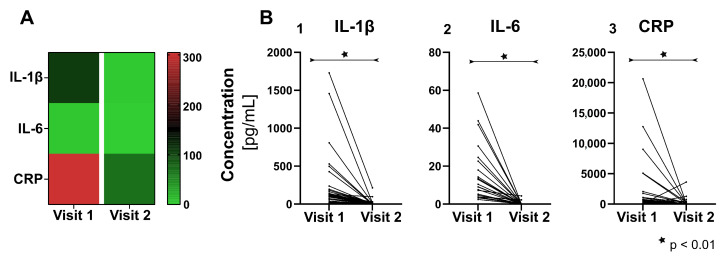
Changes in the concentration of the cytokines Interleukin (IL)--1β, IL-6, and C-reactive protein (CRP) between visit 1 and visit 2 for all groups. (**A**) Heatmap giving an overview over the medians and (**B**) diagrams on the single-sample level for concentrations of IL-1β (graph 1 of Figure 2B), IL-6 (Graph 2) and CRP (Graph 3).

As shown in Figure 2, which considers all complete datasets without differentiation between groups, concentrations of IL-1β, IL-6 and CRP a significant reduction (p_i_ < 0.001, Table S3) was found in visit 2 (medians: C_IL-1β_ = 2.27 pg/mL, C_IL-6_ = 0.00 pg/mL, C_CRP_ = 67.76 pg/mL) compared to visit 1 (medians: C_IL-1β_ = 107.35 pg/mL, C_IL-6_ = 3.81 pg/mL, C_CRP_ = 304.89 pg/mL). Within the single intervention groups (GP, BD, PB, SE) the changes remained significant in all groups for IL-1β (p_i_ < 0.05, Table S3), whereas for IL-6 and CRP concentrations at visits 1 and 2 were significantly reduced in GP and BD (p_i_, IL-6; CRP < 0.05; Table S3), but were not significantly different with PB and SE (p_i_, IL-6 ≥ 0.068; p_i_, CRP ≥ 0.09; Table S3).

### 3.3. Proteins of Tissue Homeostasis

**Figure 3:**
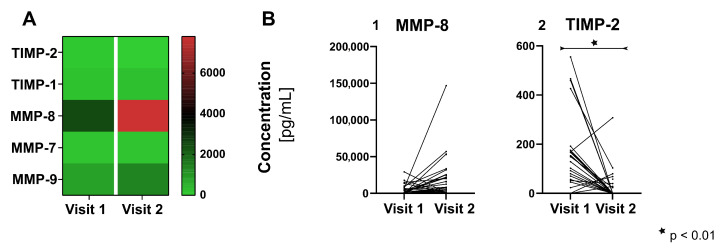
Changes in the concentrations of the matrix metalloproteinases (MMP) and tissue inhibitors of metalloproteinases (TIMP) from visit 1 to visit 2 for all groups. (**A**) Heatmap giving an overview over the medians and and (**B**) diagrams on the single-sample level for concentrations of MMP-8 (Graph 1 of Figure 2B) and TIMP-2 (Graph 2).

MMP-8 showed a significant increase in the concentration in visit 2 (median: C_MMP-8_ = 7805.4 pg/mL) compared to visit 1 (median: C_MMP-8_ = 2481.63 pg/mL, *p* = 0.016), including all intervention groups (Figure 3A,B, Table S4). Considering the single intervention groups, there was no statistical significance, but the trend remained the same with an increase of MMP-8 concentration from visit 1 to visit 2 (p_i_ ≥ 0.161, Table S4). For TIMP-2 the concentration at visit 1 (median: C_TIMP-2_ = 86.04 pg/mL) was significantly higher than at visit 2 (median: TIMP-2 = 0 pg/mL) (*p* < 0.001, Figure 3A,B, Table S5). For GP, BD and SE this effect remained significant for the single group (p_i_ < 0.46, Table S5). TIMP-1, MMP-7 and MMP-9 did not show significant changes in the concentrations between both visits (Tables S4 and S5).

**Figure 4:**
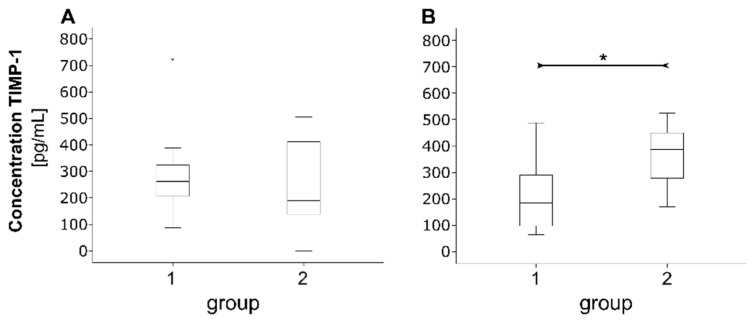
Boxplots showing the concentration of TIMP-1 concentration in group 1 (BD, GP) and group 2 (PB, SE) at visit 1 (**A**) and visit 2 (**B**). * Mann–Whitney U test (*p* = 0.064).

For TIMP-1, no statistically verifiable difference was found between groups at visit 1 (*p* > 0.05, Figure 4A). At visit 2, a trend of lower concentration in group 1 (BD, GP; 222.6 ± 141.9 pg/mL) compared to group 2 (PB, SE; 357.3 ± 121.1 pg/mL, *p* = 0.064, Figure 4B) was observed.

## 4. Discussion

Treatment of deep dentin carious lesions is a challenge. Thereby selective carious tissue removal is recommended as a treatment strategy in cases without pulpal symptoms. Some questions remain in this context: What has already happened to the pulp-dentin complex, and are there markers for inflammation which predict irreversible inflammatory changes? How do different therapeutic interventions (e.g., direct application of a dental adhesive or covering deep dentin with a liner) influence the inflammatory status of the pulp-dentin-complex?

This feasibility study revealed that the concentrations of IL-1β, IL-6, CRP, MMP-7, -8, -9, TIMP-1, and -2 can be quantified in cavities after SCT by the presented non-invasive method. Thus, the main hypothesis could be accepted. A significant decrease of CRP, IL-1β, and IL-6 from visit 1 to visit 2 was found, irrespective of the therapy strategy. TNF-α, IFN-γ, and IL-10 were below the detection threshold in most samples. Subsequently, they are not suited to investigate inflammation in the present study set-up under given conditions and with the applied methods. MMP-8 revealed an increase eight weeks after SCT. TIMP-1 showed a tendency of higher concentration eight weeks after SCT and direct application of the mild self-etch adhesive compared to covering the residual carious dentin.

As has been stated out by other authors, studies are needed which deliver longitudinal data considering possible predictive markers of pulp inflammation within the dentinal fluid [9]. Most in vivo studies deal with pulp tissue (supernatant) and blood, distinguishing between normal and irreversibly inflamed pulps [4,9]. The sampling method is very invasive, and the possibilities to measure longitudinal data and SCT success is limited. Dentinal fluid seems to be suited as sample material, which is easy and non-invasively to extract. Thus, it may offer a method to provide longitudinal data of cytokine concentration. However, the differences in the sampling method should be kept in mind when the results are discussed in the context of the available literature.

The present study results indicate that CRP, IL-1β, and IL-6 are cytokines measurable by the applied study set-up, sampling method, and used ELISA assays. Modern customize kits provide possibilities to analyze different cytokines with one assay managing small sampling volumes. However, the analytes TNF-α, IFN-γ, and IL-10 do not seem to be measurable with the used methodology. In pulp tissue and blood, other authors found measurable levels of TNF-α, which seemed to be different between healthy pulps and irreversible pulpitis [38,39,40]. However, the concentration in histologically healthy pulps, which was found by Abd-Elmeguid et al. [39], was 0.00 pg/mg protein, revealing quite low levels of this cytokine. In the present study, TNF-α was not measured directly in the pulp, and considered pulps did not show clinical symptoms of inflammation. Thus, concentration might not reach sufficient levels in the present samples to be detected. An in-vitro study working on extracted molars, from which dentinal fluid was extracted, found IL-1β, TNF-α, IL-6, IL-8, IL-12(p70), and IL-10 to be contained in appropriate mass range to be analyzed by MALDI-TOF assessment [41]. However, the sampling method, as well as processing and analysis, were completely different than in the present study set-up. IL-10 acts as an anti-inflammatory cytokine that may be protective in terms of pulpitis development, decreasing IL-6 release, inhibiting Th1 and Th2 immune response, and thus, limits the intensity of inflammatory reaction [3,42]. Elsahy et al. [38] showed an increase in IL-10 in teeth with caries-exposed pulps and irreversible pulpitis compared to healthy teeth. Renard et al. [43] confirmed these findings in experimentally induced pulpitis in rats. Again, IL-10 concentration was measured directly within the pulp [38,43]. Subsequently, as explained above for TNF-α, a low concentration level in carious dentin might lead to failure in measuring this cytokine.

The decrease in the concentration of CRP, IL-1β, and IL-6 between visit 1 and visit 2 can be interpreted as a decline in inflammation after the reduction of the microbial challenge by SCT. This is in accordance with the findings of other authors that IL-6 concentration is lower in healthy pulps (below detection threshold) compared with inflamed pulps/periapical lesions [39,44]. For IL-1β other authors [39] also report measurable levels in pulp tissues even in the healthy pulp, but found significantly higher concentrations in irreversible pulpitis. Also, in the stimulation of fibroblasts obtained from either healthy teeth or by pulpectomy, due to irreversible pulpitis, higher IL-1β release was found in the case of irreversible pulpitis [45].

One study [10] focused on the analysis of dentinal fluid considered neutrophil gelatinase (MMP-9) and found detectable levels in seven out of 16 samples from teeth with pulpits. Detection was not possible, due to MMP-9 levels below the detection limit in the case of necrotic pulps, as well as no clinical signs of pulpitis. The sampling method was different compared with the present study, and may be the reason that MMP-9 levels were above the detection limit in all samples of the current study. No differences between the visits could be found for MMP-9, as well as MMP-7. In contrast, MMP-8, an endopeptidase known to be highly effective in dentin degradation [37,46], was found to be the only analyte within the present study, which showed a significant increase of concentration in visit 2 compared to visit 1, irrespective of the intervention.

MMPs play an important role in dentin destruction in carious lesions [46]. Furthermore, MMPs were shown to degrade incompletely encapsulated collagen of the hybrid layer, thus interfering with adhesive bonding [37]. In this context, MMPs that are bound in the dentin, rather than from the pulp, are the major source for MMP activity in carious lesions [47]. The results of the current study revealed that after SCT, dentin degradation might increase, due to MMP-8. Other authors described a lower expression of MMP-8 in visit 2 after SCT and sealing caries altered dentin [48], as well as a slight decrease of MMP-8 activity with “increasing depth of dentinal caries lesions” [47]. Tissue inhibitors of metalloproteinases, which were also considered within the present investigation, are known to control MMP activity, thus limiting dentin breakdown can be assumed [49,50]. The results do not indicate an increase of TIMPs eight weeks after SCT; thus, no compensation of elevated MMP-8 concentration was revealed. However, in visit 2, significantly higher TIMP-1 concentration was found when remaining carious dentin was sealed directly by a mild self-etch adhesive compared to simple coverage. This confirms findings showing that mild self-etch adhesives (e.g., Clearfil SE Bond in this study) can stimulate biological processes, such as odontoblast activity, which lead to the formation of reactionary dentin [13].

A main strength of the current study is that it was conducted in vivo and observed interventions longitudinally. The presented methods allow measurement of cytokines, MMPs, and TIMPs in SCT without opening the pulp. Treatment strategies were compared, and different inflammatory and tissue homeostasis markers were investigated within a rinsing liquid of cavities. The main methodological limitations are that cavity size, as well as residual dentin thickness to the pulp, could be standardized to a limited extent. In the next step, the presented method will be further standardized considering the extraction of defined sample volumes and laboratory processes. Cytokine concentration may also be affected by less dentin area exposed during the second visit compared to the first one. Therefore, another point worth discussing, and standardizing in further research, is the area of dentin that is included in sampling for cytokine and growth factor analysis. Statistically, sample sizes within single intervention groups need to be further increased, as pooling samples appears to be problematic, due to the high variance in concentrations. This becomes particularly apparent with respect to MMP-8, where the statistical significance of the difference between visits 1 and 2 was lost at the single group level. Conclusively, comparison between interventions, in particular, group 1 (application of a substance on residual carious dentin close to the pulp before application of adhesive) and group 2 (direct application of a self-etch adhesive), were considered carefully within the current study. Another point to be discussed is that materials were chosen with respect to the investigation of antibacterial efficiency when used in SCT, as described in the respective publications [23,35]. Thereby, in group 1, gutta-percha, which is otherwise not used as a cavity liner in a clinical setting, served as a control for BD, due to its negligible effect against bacteria, biocompatibility, and inert character [23,51,52]. In the future, prospective long-term in vivo studies that consider changes in cytokine concentrations as the primary outcome are necessary to compare the success of different variants of SCT with other treatment regimens (e.g., partial pulpotomy or direct capping with MTA or tricalcium silicate cement), also taking into account clinical or radiological symptoms of pulp inflammation. Due to the reason that the method used in this study does not differentiate between active and inactive forms of MMPs, conclusions should be drawn carefully as comparability with results considering the active forms of MMPs is limited. Furthermore, the knowledge about how to interpret our results is limited, because we do not know for certain that we measured pulpal inflammation.

## 5. Conclusions

Methodologically, the present study shows that the non-invasive determination of biomarkers considering pulpal diagnostics gained from preparation cavities is possible. It can be concluded that eight weeks after SCT, fewer inflammatory cytokines were detected, regardless of whether a mild self-etch adhesive was placed directly on the dentin near the pulp or an additional protective layer was applied. SCT and possible adjunctive effects of pulp protection materials should be investigated considering the success in terms of pulp vitality and restoration success in prospective long-term studies. Further standardization of the sampling method is reasonable. Furthermore, not only protein, but also gene expression levels might be worth being investigated in future research.

## Figures and Tables

**Table 1:** Demographic and clinical characteristics of study participants and teeth. Guttapercha (GP), Biodentine^TM^ (BD), Clearfil^TM^ Protect Bond (PB), Clearfil^TM^ SE Bond 2 (SE).

	Group 1 (GP and BD) ^1^ (*n* = 11)		Group 2 (PB and SE) ^2^ (*n* = 10)	
**Sex, *n* (%)**				
**Males**	8 (72.7)		7 (70.0)	
**Females**	3 (27.3)		3 (30.0)	
**Median age, y (min–max)**	26 (19–40)		23.5 (19–39)	
	**GP**	**BD**	**PB**	**SE**
**Tooth type, *n* (%)**				
**Premolars**	8 (72.7)	7 (63.6)	2 (20)	5 (50)
**Molars**	3 (27.3)	4 (36.4)	8 (80)	5 (50)
**Restorations, *n* (%)**				
**Class I**	1 (9.1)	1 (9.1)	0 (0)	0 (0)
**Class II**	10 (90.9)	10 (90.9)	10 (100)	10 (100)
**Carious dentin consistency**				
**visit I, *n* (%)**				
**soft**	5 (45.5)	2 (18.2)	2 (20)	8 (80)
**medium**	3 (27.3)	7 (63.6)	8 (80)	2 (20)
**hard**	3 (27.3)	2 (18.2)	0 (0)	0 (0)
**Carious dentin consistency**				
**visit II, *n* (%)**				
**soft**	3 (27.3)	1 (9.1)	0 (0)	0 (0)
**medium**	2 (18.2)	4 (36.4)	9 (90)	7 (70)
**hard**	6 (54.5)	6 (54.5)	1 (10)	2 (20)
**Postoperative symptoms, *n* (%)**	0 (0)	0 (0)	0 (0)	1 (10)
**Drop-outs, *n* (%)**	0 (0)	0 (0)	0 (0)	1 (10)

^1^ [23], ^2^ [35].

## Data Availability

The data presented in this study are available on request from the corresponding author. The data are not publicly available due to the reason that our ethics committee does not agree with a generalized transfer of the data.

## Supplementary Materials

The following are available online at https://www.mdpi.com/2077-0383/10/5/1003/s1, Figure S1: Consort diagram considering patient recruitment and inclusion in the study, Figure S2: Modified consort diagram giving an overview over the number of patients included into the studies and the reasons for missing data of the different assays, Table S1: Materials and devices used within the study, Table S2: Quantitative evaluation of pro-inflammatory cytokines when all groups were pooled and differentiated for the single interventional groups, Table S3: Quantitative evaluation of pro-inflammatory cytokines when all groups were pooled and differentiated for the single interventional groups, Table S4: Quantitative evaluation of matrix metalloproteinases when all groups were pooled and differentiated for the single interventional groups, Table S5: Quantitative evaluation of tissue inhibitor of metalloproteinases when all groups were pooled and differentiated for the single interventional groups.
