# Supplementary material for: Detection of Inflammatory and Homeostasis Biomarkers after Selective Removal of Carious Dentin—An In Vivo Feasibility Study
Source: J Clin Med. 2021 Mar 2;10(5):1003. doi: 10.3390/jcm10051003 (PMC7958315; doi:10.3390/jcm10051003)
Supplement: Supplementary file 1 [file jcm-10-01003-s001.pdf]

Table S1. Materials and devices used within the study.

Material	Manufacturer	Composition	Preparation/Application Procedure
Biodentine™	Saint-Maur-des-Fossés Cedex, France	***Powder:*** Tricalcium silicate, zirconium oxide, calcium oxide, calcium carbonate, and colourings.<br>***Aqueous solution:*** Calcium chloride and polycarboxylate.	1) Gently tapping the capsule on a hard surface<br>2) Pouring of 5 drops from single-dose container into the capsule<br>3) Closing of capsule, placing capsule into mixing device (Cap-Mix) for 30 seconds
SDR™	Dentsply DeTrey, Konstanz, Germany	Barium-alumino-fluoroborosilicate glass, strontiumalumino-fluoro-silicate glass, modified urethane, dimethacrylate resin, EBPADMA, TEGDMA, CQ, photoaccelerator, BHT, UV stabilizer, titanium dioxide, iron oxide pigments, fluorescing agent	1) Dispense SDR™ in layers of maximum 4 mm<br>2) Light-curing for at least 20 seconds per layer
CeramX™	Dentsply DeTrey, Konstanz, Germany	Ethoxylated Bisphenol A Dimethacrylate, Urethane modified Bis-GMA dimethacrylate resin, 2,2′-ethylenedioxydiethyl dimetharcylate, ytterbium trifluoride, 2,6-di-tert-butyl-p-cresol	1) Application of material in increments up to a thickness of 2 mm<br>2) Light-curing of each increment for at least 20 seconds<br>3) Finishing and polishing
Guttapercha Beefill Cartridge	VDW Dental, Munich, Germany	Zinc oxide, guttapercha, barium, sulfate, wax, composites, colourings	1) Application of thermo-plasticized guttapercha on a spatula<br>2) Rapid adaption on the residual carious dentin nearby the pulp
Optibond™ FL	Kerr GmbH, Biberach, Germany	***Primer:*** Ethanol, ethylethanol, 2-Hydroxyethylmethacrylate, GPDM, PAMA, water, Camphorquinone<br>***Bond:*** Bisphenol-A-dimethacrylate, 2-Hydroxyethylmethacrylate (HEMA), Triethylenglycoldimethacrylate, 3-Methacrylolyl-oxypropyltrimethoxy-silane, Ba-Al silica glass, Camphorquinone, Na_2SiF_6	1) Etching with phosphoric acid (37%) for 20 seconds<br>2) Rinsing with water for 15 seconds<br>3) mild air flow for drying for 5 seconds<br>4) Gently application of primer for 15seconds<br>5) mild air flow for 5 seconds<br>6) Application of bond<br>7) Light curing for 30 seconds
Clearfil™ SE Bond 2	Kuraray Noritake Dental Corporation, Okayama, Japan	***Primer:*** MDP, HEMA, Hydrophobic dimethacrylate. N-Diethanol-p-toluidine, amphorquinone, water<br>***Bond:*** MDP, Bis-GMA, HEMA, silanated colloidal silica, hydrophobic dimethacrylate, Camphorquinone, water, N-Diethanol-p-toluidine	1) Gently application of primer for 20 seconds<br>2) Mild air flow for drying for 10 seconds<br>3) Application of bond<br>4) Gently air flow<br>5) Light curing for 10 seconds
Clearfil™ Protect Bond	Kuraray Noritake Dental Corporation, Okayama, Japan	***Primer:*** water, MDP, MDPB, HEMA, hydrophobic methacrylate<br>***Bond:*** MDP, DEMA, Bis-GMA, hydrophobic dimethacrylate, di-Camphorquinone, N-Diethanol-p-toluidine, silanated colloidal silica	1) Gently application of primer for 20 seconds<br>2) Mild air flow for drying for 10 seconds<br>3) Application of bond<br>4) Gently air flow<br>5) Light curing for 10 seconds
Opaldam	Ultradent Products, South Jordan, USA	Cetylalkohol, Diurethan-dimethacrylate	1) Application of the material in case of problems to seals the cavity with the matrix properly<br>2) Light-curing for at least 20 seconds
PBST (10X)	G-Biosciences GmbH, St. Louis, USA	80mM Na 2 HPO 4, 1.5M NaCl, 20mM KH 2 PO 4, 30mM KCl, 0.5% Tween® 20, pH 7.4.	1) Filling the prepared cavity with PBST<br>2) Incubating for 5 minutes

	3) Regaining of fluid with a 200 µl microtiter pipette

Table S2. Quantitative evaluation of pro-inflammatory cytokines when all groups were pooled and differentiated for the single interventional groups: guttapercha (GP), Biodentine (BD), Clearfil Protect Bond (PB), Clearfil SE Bond 2 (SE) (median, maximum, minimum; concentration, pg/mL).

LegendPlex Panel (BioLegend GmbH, Koblenz, Germany)	Analysis of IL-1β, IL-6, IL-10, CRP, TNF-α, IFN-γ, TIMP-1, TIMP-2

- preparation of the masterplate (96 well plate), dilutions were performed on second 96 well plate (1:1 dilution, sample volume 30µl + assay buffer 30µl)
- preparation of wash buffer: 47.5 mL deionized water + 2.5 mL wash buffer 20X
- preparation of matrix: lyophilized matrix + 5 mL assay buffer (vortex!)
- preparation of standard: lyophilized standard cocktail + 250 µL assay buffer
  - standard = C7, labelling of tubes C7- C0
  - addition of 75 µL of assay buffer to C6-C1, preparation of 1:4 dilution transferring 25µl of the standard

The entire assay was performed on a filter-well plate.

- addition of 100 µL wash buffer to filter plate, let sit for 1 min at room temperature
- centrifugation for 30 seconds at 300 rpm, flow through discarded
- addition of 25 µL of assay buffer to the sample wells
- addition of 25 µL of matrix to the standard wells
- addition of 25 µL of each standard to the standard wells
- addition of 25 µL of each diluted sample to the sample wells and vortex
- addition of 25 µL beads to each well (volume should be 75 µL in each well)
- overnight on a plate shaker (500 rpm 4 °C), sealing the plate with aluminium foil
- next day: centrifugation 30 seconds, 300 rpm, flow through discarded
- addition of 200 µL wash buffer to each well and centrifugation 30 seconds, 300 rpm to remove wash buffer, repetition
- addition of 25 µL detection antibodies (biotinylated) to each well, sealing the plate with aluminum foil
- shaking for 30 min 500 rpm at room temperature
- addition of 25 µL of SA-PE to each well and sealing the plate with aluminium foil
- shaking for 30 min 500 rpm at room temperature on a plate shaker, centrifugation 30 seconds at 300 rpm, flow through discarded
- addition of 200 µL wash buffer to each well, centrifugation 30 seconds at 300 rpm to remove wash buffer, repetition
- addition of 150 µL wash buffer to each wellse
- reading samples on flow cytometer with 300 events per cytokine, thus 1500 events

Human Custom 3-Plex assay (Aimplex Biosciences, Inc., Pomona, USA)	Analysis of MMP-7, -8, -9

- preparation of wash buffer: 135 mL deionized water + 15 mL wash buffer 10X
- preparation of reading buffer: 45 mL deionized water + 5 mL reading buffer 10X
- preparation of the lyophilized standard: 250 µL standard diluent + lyophilized standard (vortex for 15 seconds), incubation on ice for 10 min
  - serial dilution preparation:
  - standard = C7, labelling of tubes C7- C0
  - addition of 160 µL of standard diluent to C6-C1, preparation of 1:3 dilution transferring 80µl of the standard

The entire assay was performed on a filter-plate.

- preparation of filter plate: wash with 100 µL wash buffer, centrifugation at 300 rpm for 30 seconds, flow-through discarded
- addition of 45 µL bead suspension to each well, centrifugation at 300 rpm for 30 seconds, flow-through discarded
- addition of 30 µL assay buffer to each sample well
- addition of 15 µL of samples (no dilution!) to each sample well
- addition of 45 µL standard to each standard well
- sealing the plate with aluminium foil
- overnight incubation on a plate shaker (500 rpm at 4 °C)
- washing the wells 3 times with 100 µL wash buffer and centrifugation at 300 rpm for 30 seconds, flow-through discarded after each washing step
- addition of 25 µL of biotinylated antibody working solution to each well
- sealing the plate with aluminium foil
- shaking at 500 rpm for 1 hour at room temperature
- washing the wells 3 times with 100 µL wash buffer and centrifugation at 300 rpm for 30 seconds, flow-through discarded
- addition of 25 µL of SA-PE to each well and sealing the plate with aluminium foil

- shaking at 500 rpm for 30 min at room temperature
- washing the wells twice with 100 µL wash buffer, centrifugation at 300 rpm for 30 seconds, flow-through discarded after each washing step
- addition of 150 µL reading buffer and covering the plate with a plate seal
- reading samples on flow cytometer with 100 events per cytokine, thus 300 events

SA-PE: Streptavidin-Phycoerythrin

Table S3. Quantitative evaluation of pro-inflammatory cytokines when all groups were pooled and differentiated for the single interventional groups: guttapercha (GP), Biodentine (BD), Clearfil Protect Bond (PB), Clearfil SE Bond 2 (SE) (median, maximum, minimum; concentration, pg/mL).

	**Median (Min – Max), pg/mL**								
	IL-1β *visit 1*	IL-1β *visit 2*	$p^{\dagger}$	IL-6 *visit 1*	IL-6 *visit 2*	$p^{\dagger}$	CRP *visit 1*	CRP *visit 2*	$p^{\dagger}$
**all groups**	107.35 (0.96 – 1728.60)	2.27 (0.00 – 213.59)	**<0.001**	3.81 (0 – 58.54)	0.00 (0.00 – 4.33)	**<0.001**	304.89 (0.00 – 20626.52)	67.76 (0.00 – 3628.34)	**<0.001**
**GP**	111.10 (0.96 – 1728.60)	8.45 (0.00 – 213.59)	**0.008**	4.71 (0.00 – 41.88)	0.00 (0.00 – 4.33)	**0.028**	377.67 (0.00 – 12752.43)	85.87 (0.00 – 1287.02)	**0.041**
**BD**	115.98 (1.32 – 1456.96)	2.14 (0 – 97.14)	**0.007**	3.81 (0.00 – 14.31)	0.00 (0.00 – 2.21)	**0.012**	1018.74 (15.96 – 20626.52)	98.01 (18.05 – 3628.34)	**0.047**
**PB**	103.59 (3.06 – 496.85)	1.08 (0.00 – 12,63)	**0.018**	0.00 (0.00 – 43.90)	0.00 (0.00 – 0.00)	0.109	219.21 (18.05 – 1019.85)	67.76 (0.00 – 438.42)	0.091
**SE**	66.51 (5.95 – 237.22)	0.00 (0.00 – 21.03)	**0.028**	6.49 (0.00 – 58.54)	0.00 (0.00 – 0.00)	0.068	131.13 (44.68 – 788.36)	39.90 (0.00 – 273.4)	0.091

†Wilcoxon test

Table S4. Quantitative evaluation of matrix metalloproteinases when all groups were pooled and differentiated for the single interventional groups: guttapercha (GP), Biodentine (BD), Clearfil Protect Bond (PB), Clearfil SE Bond 2 (SE) (median, maximum, minimum; concentration, pg/mL).

	**Median (Min – Max), pg/mL**								
	MMP-7 *visit 1*	MMP-7 *visit 2*	$p^{\dagger}$	MMP-8 *visit 1*	MMP-8 *visit 2*	$p^{\dagger}$	MMP-9 *visit 1*	MMP-9 *visit 2*	$p^{\dagger}$
**all groups**	141.42 (52.83 – 547.36)	155.56 (60.61 – 653.22)	0.122	2481,63 (72.67 – 29168.48)	7805.40 (26.46 – 146831.88)	**0.016**	822.15 (46.79 – 108743.19)	1363.22 (43.71 – 176680.20)	0.264
**GP**	141.42 (78.08 – 435.72)	307.02 (73.18 – 653.22)	0.214	3694.43 (374.34 – 17979.70)	8488.92 (270.48 – 146831.88)	0.173	413.46 (68.62 – 108743.19)	818.44 (49.89 – 176680.20)	0.314
**BD**	123.88 (52.83 – 247.92)	126.13 (60.61 – 291.18)	0.263	641.09 (72.67 – 13570.06)	7495.00 (26.46 – 53568.46)	0.161	648.67 (46.79 – 3988.13)	285.14 (43.71 – 31259.98)	0.327
**PB**	195.11 (75.64 – 519.7)	200.84 (136.42 – 515.07)	0.612	3046.79 (97.41 – 17278.35)	12535.42 (113.24 – 52973.52)	0.176	3526.33 (78.05 – 30313.80)	6923.04 (193.92 – 35758.05)	0.499
**SE**	148.79 (68.21 – 547.36)	156.15 (99.37 – 539.64)	0.735	1989.71 (233.24 – 29168.48)	3192.24 (208.54 – 25914.02)	0.866	1828.00 (193.92 – 29148.36)	1291.75 (100.11 – 14007.14)	0.499

†Wilcoxon test

Table S5. Quantitative evaluation of tissue inhibitor of metalloproteinases when all groups were pooled and differentiated for the single interventional groups: guttapercha (GP), Biodentine (BD), Clearfil Protect Bond (PB), Clearfil SE Bond 2 (SE) (median, maximum, minimum, pg/mL).

	**Median (Min – Max), pg/mL**					
	TIMP-1 *visit 1*	TIMP-1 *visit 2*	$p^{\dagger}$	TIMP-2 *visit 1*	TIMP-2 *visit 2*	$p^{\dagger}$
**all groups**	198.6 (0.00 – 1011.16)	245.80 (0.00 – 683.49)	0.688	86.04 (0.00 – 555.35)	0.00 (0.00 – 307.63)	**< 0.001**
**GP**	267.04 (0.00 – 961.35)	205.22 (0.00 – 683.49)	0.799	70.29 (0.00 – 466.35)	0.00 (0.00 – 103.44)	**0.036**
**BD**	198.60 (0.00 – 615.66)	160.32 (0.00 – 580.35)	0.799	141.05 (0.00 – 555.35)	0.00 (0.00 – 307.63)	**0.038**
**PB**	155.99 (0.00 – 823.05)	462.96 (133.52 – 556.26)	0.237	70.29 (0.00 – 175.48)	0.00 (0.00 – 68.16)	0.080
**SE**	207.18 (0.00 – 1011.16)	329.18 (0.00 – 568.38)	0.463	68.31 (0.00 – 191.92)	0.00 (0.00 – 28.49)	**0.046**

† Wilcoxon test
